# Supplementary material for: In search of nonlipogenic ABCA1 inducers (NLAI): precision coregulator TR-FRET identifies diverse signatures for LXR ligands
Source: bioRxiv. 2025 Sep 21:2025.09.12.675753. Preprint. [Version 2] doi: 10.1101/2025.09.12.675753 (PMC12458303; doi:10.1101/2025.09.12.675753)
Supplement: Supplement 1 [file media-1.pdf]

In search of nonlipogenic ABCA1 Inducers (NLAI): precision coregulator TR-FRET identifies diverse signatures for LXR ligands

Megan S. Laham[b], Martha Ackerman-Berrier[a], Fahmida Alam[b], Sarah Turner[a], Ganga Reddy Velma[a], Christopher Penton[a], Soumya Reddy Musku[a], Manan Rana[a], Senthil Kumar[a], Anandhan Annadurai[a], Maha Ibrahim Sulaiman[a], Nina Ma[a], Gregory R J Thatcher[a,b]*

[a]Department of Pharmacology & Toxicology, R Ken Coit College of Pharmacy, University of Arizona, Tucson, Arizona 85721, USA

[b]Department of Chemistry & Biochemistry, Colleges of Science & Medicine, University of Arizona, Tucson, AZ, 85721

[c] Arizona Center for Drug Discovery, University of Arizona, Tucson, Arizona 85721, USA

*Corresponding author: Gregory Thatcher, grjthatcher@arizona.edu

Table of Contents

1. Table S1...Page 2
2. Table S2……..……………...………………………………………………………...Page 2
3. Figure S1…..Page 3
4. Figure S2…..Page 4
5. Figure S3…..Page 4
6. Figure S4…..Page 4
7. Figure S5…..Page 5
8. Figure S6…..Page 5
9. Figure S7…..Page 6
10. Figure S8…..Page 6
11. Experimental Details…...Page 7
12. Chemical Synthesis Details….. Page 13
13. NMR Spectra…...Page 15

Table S1. Summary of NR Panel Parameters.

Lanthascreen		
**Receptor**	**Coactivator/Acceptor**	**Donor**
FXR	SRC2-2-fluorescein	α-GST-Terbium
PXR	SRC1-4-fluorescein	α-GST-Terbium
PPARδ	C33-fluorescein	α-GST-Terbium
RXRα/β	D22-fluorescein	α-GST-Terbium
RARα	D22-fluorescein	α-GST-Terbium
RARβ	SRC2-2-fluorescein	α-GST-Terbium

Table S2. Coregulator Peptide Sequences

Vendor	Catalog No.	Coregulator	Sequence	MW (Da.)
Anaspec	AS-62152	SRC1-btn	Biotin-CPSSHSSLTERHKILHRLLQEGSPS	3026.6
Thermo Fischer	PV4386	FL-D22	Fluorescein-LPYEGSLLLKLLRAPVEEV	2499
Thermo Fischer	PV4549	FL-TRAP220/DRIP-1	Fluorescein-NTKNHPMLMNLLKDNPAQD	2554
Thermo Fischer	PV4582	FL-SRC1-4	Fluorescein-GPQTPQAQQKSLLQQLLTE	2465.9
Thermo Fischer	PV4586	FL-SRC2-2	Fluorescein-LKEKHKILHRLLQDSSSPV	2585
Thermo Fischer	PV4421	FL-PGC1a	Fluorescein-EAEEPSLLKKLLLAPANTQ	2424

Thermo Fischer	PV4606	FL-C33	Fluorescein-HVEMHPLLMGLLMESQWGA	2539
Thermo Fischer	PV4624	FL-NCoR ID2	Fluorescein-DPASNLGLEDIIRKALMGSFDDK	2865
Thermo Fischer	PV4423	FL-SMRT ID2	Fluorescein -HASTNMGLEAIIRKALMGKYDQW	2993

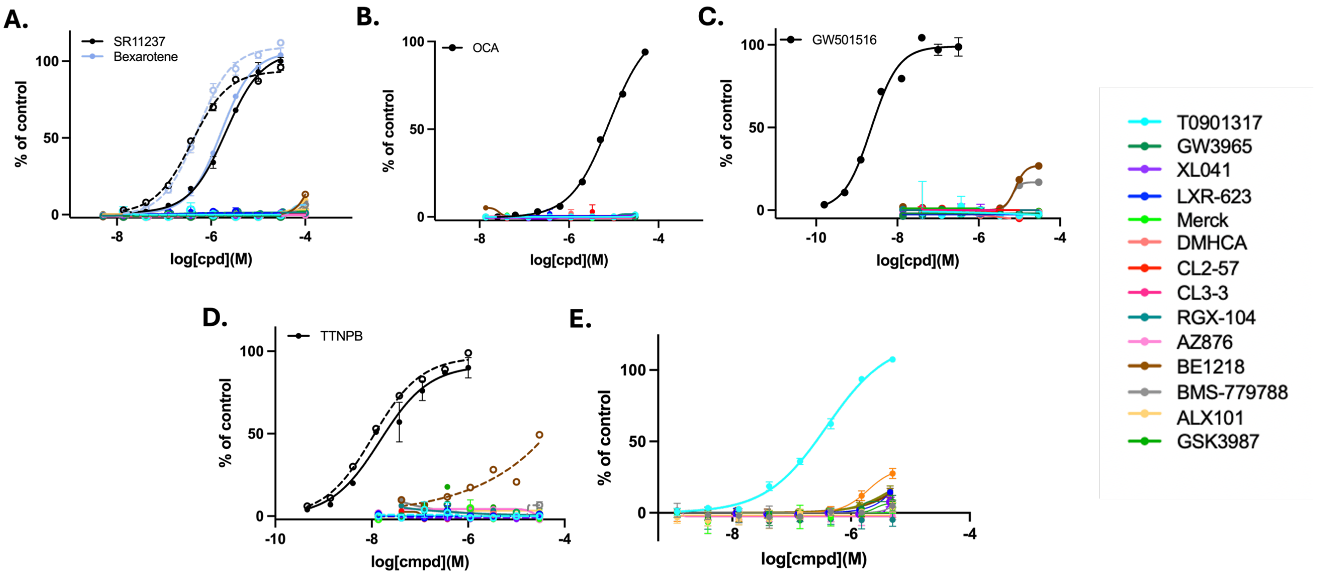

Figure S1. NR Panel Results A. Concentration-response for recruitment of coregulator D22 to RXRα (dotted lines) and RXRβ (solid line), determined by CRT assay, showing mean and SD, normalized to SR11237 as a full agonist. **B.** Concentration-response for recruitment of coregulator SRC2 to FXR determined by CRT assay, showing mean and SD, normalized to Obeticholic acid (OCA) as a full agonist. **C.** Concentration-response for recruitment of coregulator C33 to PPARδ determined by CRT assay, showing mean and SD, normalized to GW501516 as a full agonist. **D.** Concentration-response for recruitment of coregulator D22 to RARα (dotted lines) and recruitment of coregulator SRC2-2 RARβ (solid line), determined by CRT assay, showing mean and SD, normalized to TTNPB as a full agonist. **E.** Concentration-response for recruitment of coregulator SRC1-4 to PXR determined by CRT assay, showing mean and SD, normalized to T0901317(T0) as a full agonist.

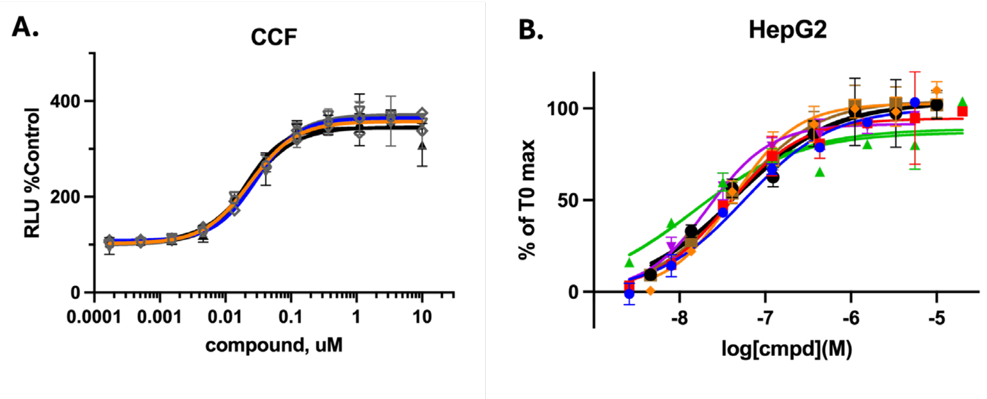

Figure S2. Concentration-response curves of the control compound, **T0**, in CCF ABCA1-luc reporter assay and HepG2 SRE-luc reporter assay (**A**, **B**) show high reproducibility across independent experiments.

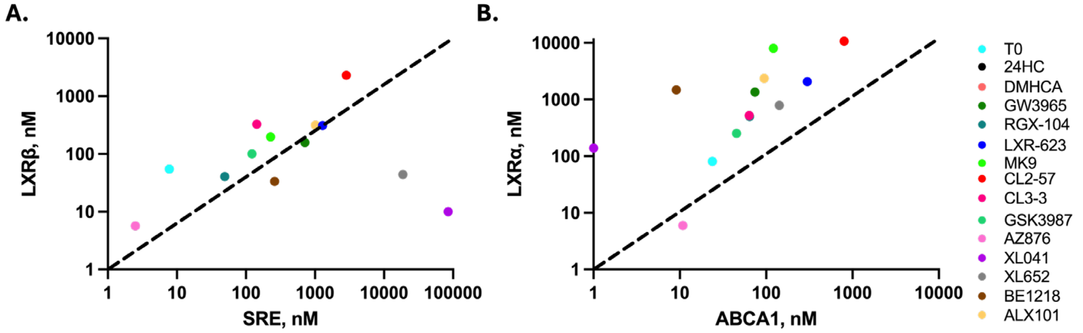

Figure S3. Additional correlation analyses of LXR isoforms with SRE-luc and ABCA1-luc reporter data.

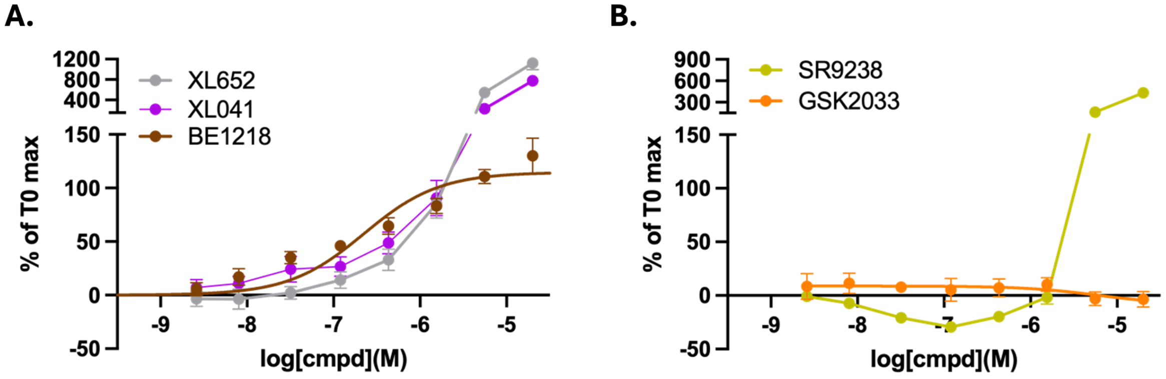

Figure S4. Lipogenic response of HepG2 cells to treatment with LXR ligands. full concentration-response curves for biphenyl compounds from **Fig. 5E-F.**

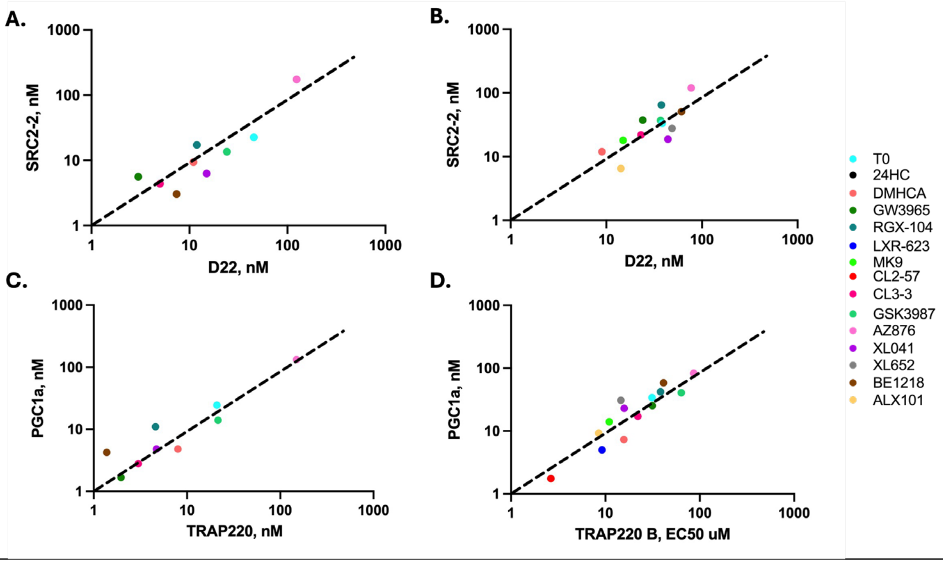

Figure S5. Correlation analyses of coactivator responses reveal consistent trends across several coactivators, hence the condensed data presentation in the main text.

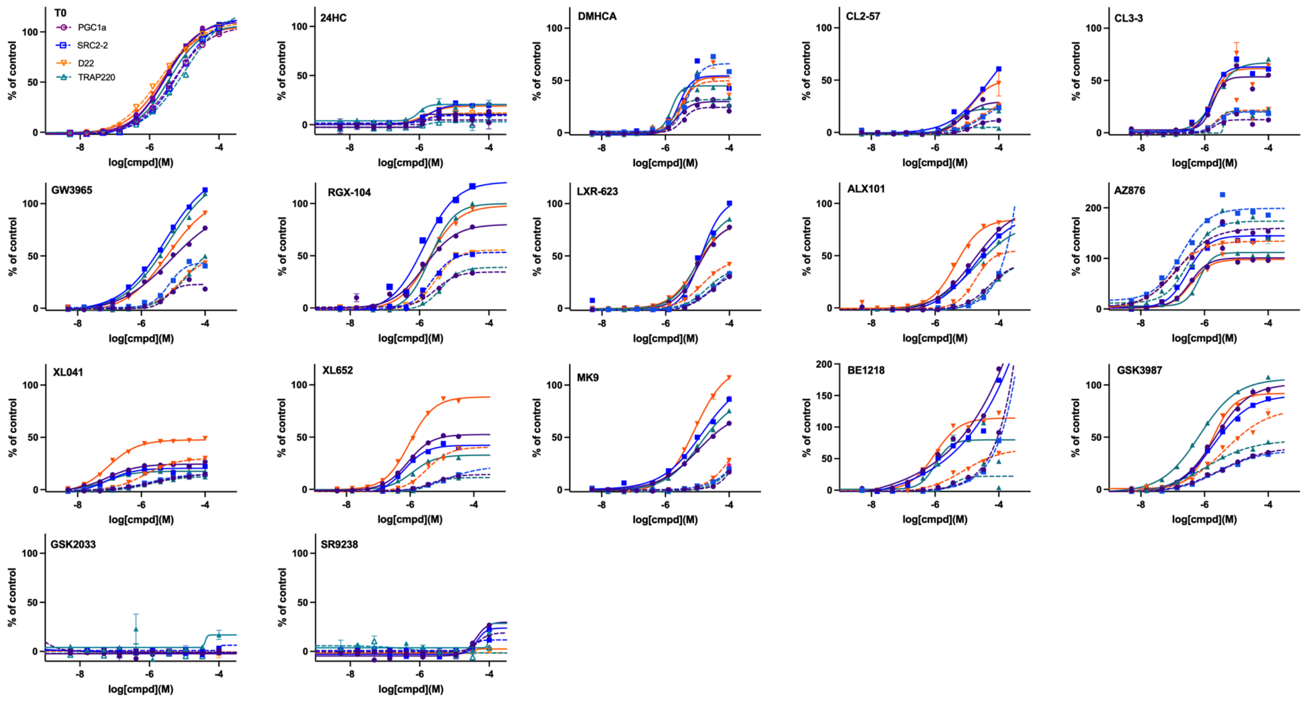

Figure S6. Full concentration-response profiles of all tested compounds across coactivators. Shown here for completeness; LXRα (dashed lines) and LXRβ (solid lines).

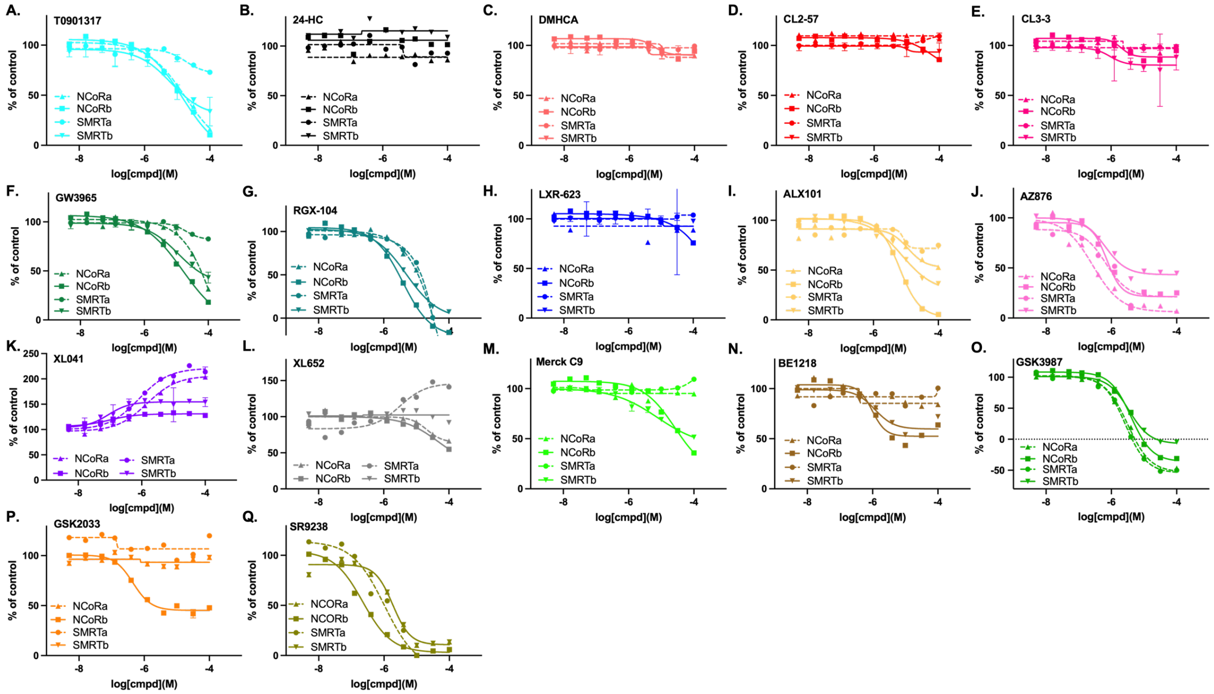

Figure S7. Full concentration-response profiles of all tested compounds across corepressors NCor2 and SMRT for both LXRα/β isoforms.

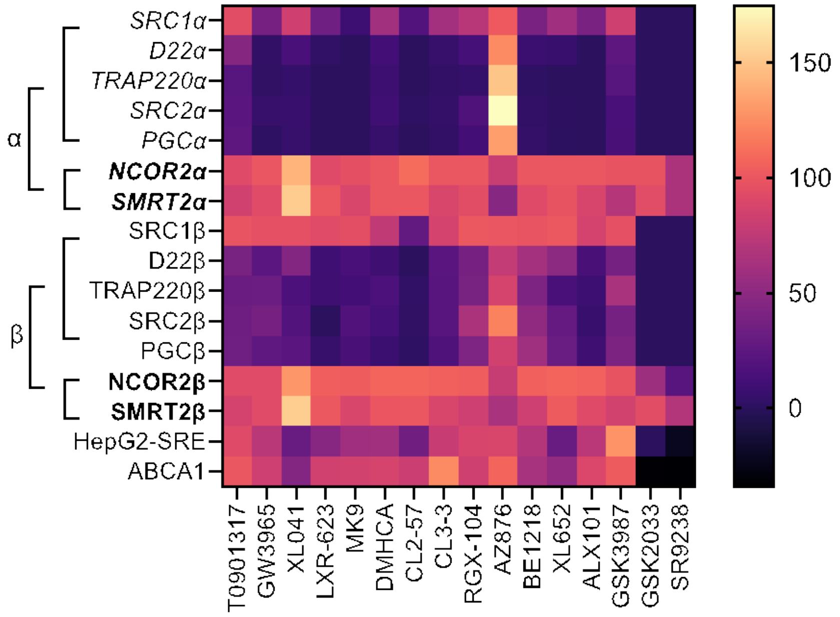

Figure S8. Heat map illustrating the range of coregulator efficacy responses across all ligands and both LXR isoforms, highlighting variability and pattern differences.

Materials and Methods:

CRT Assays

Coactivator recruitment LXRα/β HTRF

To determine compound EC50 values, 10mM compound stocks were prepared in DMSO. An initial 8-concentration, 3-fold dilution series in DMSO was performed in 96-well v-bottom, polypropylene plates at 100x the final desired concentration. Intermediate dilution plates were prepared by diluting the 100x DMSO stocks 25-fold in assay buffer (50mM Tris, pH 7.4, 50mM KCl, 0.05% Triton-X 100, 10% glycerol, 0.1% BSA- (added fresh), 5mM DTT (added fresh)), creating a 4x stock. The following assay components were prepared individually at a 4x final concentration in assay buffer: (1) LXRα/β -GST tag (Thermo Fisher Scientific PV4657/PV PV4660); (2) a mixture of anti-GST-Tb (CisBio/Perkin Elmer 61GSTTLA, lot 14A) and SA-XL665 (CisBio/Perkin Elmer 610SAXLB); (3) SRC1-btn (AnaSpec AS62152/Fisher Scientific NC1565694). A 3uL aliquot of the 4x ligand was then added to each well of the 384-well small volume non-binding black assay plate (Greiner 784900) followed by a 3uL aliquot of each reaction component for a total volume of 12uL. Control wells used contained all components minus ligands, replaced by positive control T0901317 (Enzo 270-309-M010/VWR 89147-616) or DMSO. Incubated at 1hr at room temperature before measurement of TR-FRET. Final concentrations of assay components LXRα/β (4nM), anti-GST-Tb (0.4nM), SA-XL665(9.44nM), and SRC1-btn (100nM). TR-FRET measurements were performed on a CLARIOstar® Plus multi-mode plate reader (BMG Labtech, Ortenberg, Germany), excitation 337nm (laser), emission1 615nm and emission2 665nm. The signal is expressed as a ratio of TR-FRET measurements at 665 nm/615nm × 1000. Experimental data were normalized to controls and fit with a four parameter Hill equation using Graphpad Prism software (Boston, MA) to determine EC50s.

Coactivator recruitment Lanthascreen RXRα/β

To determine compound EC50 values, 10mM compound stocks were prepared in DMSO. An initial 8-concentration, 3-fold dilution series in DMSO was performed in 96-well v-bottom, polypropylene plates at 100x the final desired concentration. Intermediate dilution plates were prepared by diluting the 100x DMSO stocks 25-fold in assay buffer (50mM Tris, pH 7.4, 50mM KCl, 0.05% Triton-X 100, 10% glycerol, 0.1% BSA- (added fresh), 5mM DTT (added fresh)), creating a 4x stock. The following assay components were prepared individually at a 4x final concentration in assay buffer: (1) RXRα/β (Thermo Fisher Scientific PV4799/ PV4390); (2) anti-GST-Tb (CisBio/Perkin Elmer 61GSTTLA, lot 14A); (3) D22-FL (Thermo Fisher Scientific PV4386). A 3uL aliquot of the 4x ligand was then added to each well of the 384-well small volume non-binding black assay plate (Greiner 784900) followed by a 3uL aliquot of each reaction component for a total volume of 12uL. Control wells used contained all components minus ligands, replaced by positive control SR11273 (Sigma-Aldrich S8951) or DMSO. Incubated at 1hr at room temperature before measurement of TR-FRET. Final concentrations of assay components RXRα/β (1nM), anti-GST-Tb (1nM), and D22-FL (200nM). TR-FRET measurements were performed on a CLARIOstar® Plus multi-mode plate reader (BMG Labtech, Ortenberg, Germany), excitation 337nm (laser), emission1 520nm and emission2 490nm. The signal is expressed as a ratio of TR-FRET measurements at 520 nm/490nm × 1000. Experimental data were normalized to controls and fit with a four parameter Hill equation using Graphpad Prism software (Boston, MA) to determine EC50s.

Coactivator recruitment Lanthascreen FXR

To determine compound EC50 values, 10mM compound stocks were prepared in DMSO. An initial 8-concentration, 3-fold dilution series in DMSO was performed in 96-well v-bottom, polypropylene plates at 100x the final desired concentration. Intermediate dilution plates were prepared by diluting the 100x DMSO stocks 25-fold in assay buffer (50mM Tris, pH 7.4, 50mM KCl, 0.05% Triton-X 100, 10% glycerol, 0.1% BSA- (added fresh), 5mM DTT (added fresh)), creating a 4x stock. The following assay components were prepared individually at a 4x final

concentration in assay buffer: (1) FXR (Thermo Fisher Scientific **PV4835)**; (2) anti-GST-Tb (CisBio/Perkin Elmer 61GSTTLA, lot 14A); (3) SRC2-2-FL (Thermo Fisher Scientific PV4586). A 3uL aliquot of the 4x ligand was then added to each well of the 384-well small volume non-binding black assay plate (Greiner 784900) followed by a 3uL aliquot of each reaction component for a total volume of 12uL. Control wells used contained all components minus ligands, replaced by positive control Obeticholic acid (Sigma-Aldrich SML3096) or DMSO. Incubated at 1hr at room temperature before measurement of TR-FRET. Final concentrations of assay components FXR (2.5nM), anti-GST-Tb (1nM), and SRC2-2-FL (250nM). TR-FRET measurements were performed on a CLARIOstar® Plus multi-mode plate reader (BMG Labtech, Ortenberg, Germany), excitation 337nm (laser), emission1 520nm and emission2 490nm. The signal is expressed as a ratio of TR-FRET measurements at 520 nm/490nm × 1000. Experimental data were normalized to controls and fit with a four parameter Hill equation using Graphpad Prism software (Boston, MA) to determine EC50s.

Coactivator recruitment Lanthascreen PPARδ

To determine compound EC50 values, 10mM compound stocks were prepared in DMSO. An initial 8-concentration, 3-fold dilution series in DMSO was performed in 96-well v-bottom, polypropylene plates at 100x the final desired concentration. Intermediate dilution plates were prepared by diluting the 100x DMSO stocks 25-fold in assay buffer ((20mM Tris, pH 7.9, 100mM KCl, 0.01% Triton-X 100, 0.1% BSA (added fresh)), creating a 4x stock. The following assay components were prepared individually at a 4x final concentration in assay buffer: (1)PPARδ (Thermo Fisher Scientific PV4694**)**; (2) anti-GST-Tb (CisBio/Perkin Elmer 61GSTTLA, lot 14A); (3) C33-FL (Thermo Fisher Scientific PV4606). A 3uL aliquot of the 4x ligand was then added to each well of the 384-well small volume non-binding black assay plate (Greiner 784900) followed by a 3uL aliquot of each reaction component for a total volume of 12uL. Control wells used contained all components minus ligands, replaced by positive control GW501516 (MedChemExpress, HY-10838/CS-0438) or DMSO. Incubated at 1hr at room temperature before

measurement of TR-FRET. Final concentrations of assay components PPARδ (5nM), anti-GST-Tb (1nM), and C33-FL (250nM). TR-FRET measurements were performed on a CLARIOstar® Plus multi-mode plate reader (BMG Labtech, Ortenberg, Germany), excitation 337nm (laser), emission1 520nm and emission2 490nm. The signal is expressed as a ratio of TR-FRET measurements at 520 nm/490nm × 1000. Experimental data were normalized to controls and fit with a four parameter Hill equation using Graphpad Prism software (Boston, MA) to determine EC50s.

Coactivator recruitment Lanthascreen RARα.

To determine compound EC_{50} values, 10mM compound stocks were prepared in DMSO. An initial 8-concentration, 3-fold dilution series in DMSO was performed in 96-well v-bottom, polypropylene plates at 100x the final desired concentration. Intermediate dilution plates were prepared by diluting the 100x DMSO stocks 25-fold in assay buffer (50 mM Tris, pH 7.4, 50 mM KCl, 0.05% Triton-X 100, 10% glycerol, 0.1% BSA- (added fresh), 5 mM DTT (added fresh)), creating a 4x stock. The following assay components were prepared individually at a 4x final concentration in assay buffer: (1) RARα (Thermo Fisher Scientific PV4799/ PV4390); (2) anti-GST-Tb (CisBio/Perkin Elmer 61GSTTLA, lot 14A); (3) D22-FL (Thermo Fisher Scientific PV4386). A 3 µL aliquot of the 4x ligand was then added to each well of the 384-well small volume non-binding black assay plate (Greiner 784900) followed by a 3 µL aliquot of each reaction component for a total volume of 12 µL. Control wells used contained all components minus ligands, replaced by positive control TTNPB (MedChemExpress HY-15682), or DMSO. Incubated at 1hr at room temperature before measurement of TR-FRET. Final concentrations of assay components RARα (3 nM), anti-GST-Tb (2 nM), and D22-FL (250 nM). TR-FRET measurements were performed on a CLARIOstar® Plus multi-mode plate reader (BMG Labtech, Ortenberg, Germany), excitation 337nm (laser), emission1 520 nm and emission2 490 nm. The signal is expressed as a ratio of TR-FRET measurements at 520 nm/490 nm × 1000.

Experimental data were normalized to controls and fit with a four parameter Hill equation using Graphpad Prism software (Boston, MA) to determine EC_{50} values.

Coactivator recruitment Lanthascreen RARβ.

To determine compound EC_{50} values, 10mM compound stocks were prepared in DMSO. An initial 8-concentration, 3-fold dilution series in DMSO was performed in 96-well v-bottom, polypropylene plates at 100x the final desired concentration. Intermediate dilution plates were prepared by diluting the 100x DMSO stocks 25-fold in assay buffer (50 mM Tris, pH 7.4, 50 mM KCl, 0.05% Triton-X 100, 10% glycerol, 0.1% BSA- (added fresh), 5 mM DTT (added fresh)), creating a 4x stock. The following assay components were prepared individually at a 4x final concentration in assay buffer: (1) RARβ (Thermo Fisher Scientific PV4799/ PV4390); (2) anti-GST-Tb (CisBio/Perkin Elmer 61GSTTLA, lot 14A); (3) SRC2-2-FL (Thermo Fisher Scientific PV4586). A 3 µL aliquot of the 4x ligand was then added to each well of the 384-well small volume non-binding black assay plate (Greiner 784900) followed by a 3 µL aliquot of each reaction component for a total volume of 12 µL. Control wells used contained all components minus ligands, replaced by positive control TTNPB (MedChemExpress HY-15682) or DMSO. Incubated at 1hr at room temperature before measurement of TR-FRET. Final concentrations of assay components RARβ (2.5 nM), anti-GST-Tb (2 nM), and SRC2-2-FL (300 nM). TR-FRET measurements were performed on a CLARIOstar® Plus multi-mode plate reader (BMG Labtech, Ortenberg, Germany), excitation 337 nm (laser), emission1 520 nm and emission2 490 nm. The signal is expressed as a ratio of TR-FRET measurements at 520 nm/490 nm × 1000. Experimental data were normalized to controls and fit with a four parameter Hill equation using Graphpad Prism software (Boston, MA) to determine EC_{50} values.

Coactivator recruitment Lanthascreen PXR. To determine compound EC_{50} values, 10 mM compound stocks were prepared in DMSO. An initial 8-concentration, 3-fold dilution series in DMSO was performed in 96-well v-bottom, polypropylene plates at 100x the final desired

concentration. Intermediate dilution plates were prepared by diluting the 100x DMSO stocks 33.3-fold in assay buffer (50 mM Hepes, pH 8.0, 50 mM NaCl, 0.01% TWEEN® 20), creating a 3x stock. The following assay components were prepared individually at a 3x final concentration in assay buffer: (1) PXR (Thermo Fisher Scientific PV4841); (2) anti-GST-Tb (Revvity 61GSTTLA) combined with SRC1-4-FL (Thermo Fisher Scientific PV4582). A 4 µL aliquot of the 3x ligand was then added to each well of the 384-well small volume non-binding black assay plate (Greiner 784900) followed by a 4 µL aliquot of each reaction component for a total volume of 12 µL. Control wells used contained all components minus ligands, replaced by positive control T0901317 (Synthesized by Ganga Reddy Velma, Thatcher Lab, University of Arizona; or Enzo 270-309-M010/VWR 89147-616) or DMSO. Final concentrations of assay components were: PXR (10 nM), anti-GST-Tb (1 nM), and SRC1-4-FL (300 nM). Plates were incubated for 20' at room temperature before TR-FRET measurements were performed on a CLARIOstar® Plus multi-mode plate reader (BMG Labtech, Ortenberg, Germany), excitation 337 nm (laser), emission1 520 nm and emission2 490 nm. The signal is expressed as a ratio of TR-FRET measurements at 520 nm/490 nm × 1000. Experimental data were normalized to controls and fit with a four parameter Hill equation using Graphpad Prism software (Boston, MA) to determine EC_{50} values.

Chemistry

(*R*)-2-chloro-4-(1'-(2-hydroxy-3-methyl-2-(trifluoromethyl)butanoyl)-[4,4'-bipiperidin]-1-yl)-*N*,*N*-dimethylbenzamide (**MK9**)

This compound was obtained using a procedure similar to the reported literature method.[1]

^{1}H NMR (400 MHz, $CDCl_3$) δ 7.14 (d, *J* = 8.5 Hz, 1H), 6.85 (d, *J* = 2.4 Hz, 1H), 6.80 (dd, *J* = 8.6, 2.4 Hz, 1H), 5.69 (s, 1H), 4.88 – 4.14 (m, 2H), 3.74 (dd, *J* = 12.7, 4.0 Hz, 2H), 3.11 (s, 3H), 2.88 (s, 3H), 2.71 (td, *J* = 12.1, 2.3 Hz, 2H), 2.44 (s, 1H), 1.90 – 1.76 (m, 4H), 1.45 – 1.16 (m, 6H), 1.11 (d, *J* = 6.4 Hz, 3H), 0.84 (s, 3H). ^{13}C NMR (101 MHz, $CDCl_3$) δ 169.14, 166.93, 152.49, 131.42, 128.74, 126.13, 123.17, 115.98, 114.44, 49.23, 46.49, 40.96, 40.58, 38.43, 34.89, 31.07, 29.48, 28.92, 28.87, 16.78.

Ethyl3-(5-chloro-3-(N-(3,4-diethoxyphenyl)-N-methylsulfamoyl)thiophene-2-carboxamido)benzoate (**CL2-27)**

This compound was obtained using procedures detailed in our previous publication.[2]

^{1}H NMR (400 MHz, $CDCl_3$) δ 10.03 (s, 1H), 7.94 (t, *J* = 1.9 Hz, 1H), 7.77 (dt, *J* = 7.8, 1.3 Hz, 1H), 7.59 (ddd, *J* = 8.2, 2.3, 1.1 Hz, 1H), 7.31 (t, *J* = 7.9 Hz, 1H), 6.63 – 6.57 (m, 2H), 6.53 (d, *J* = 8.5 Hz, 1H), 4.39 (q, *J* = 7.1 Hz, 2H), 3.87 (q, *J* = 6.9 Hz, 2H), 3.70 (q, *J* = 6.9 Hz, 2H), 3.20 (s, 3H), 1.41 (t, *J* = 7.1 Hz, 3H), 1.34 (t, *J* = 7.0 Hz, 3H), 1.26 (t, *J* = 7.0 Hz, 3H). ^{13}C NMR (101 MHz, $CDCl_3$) δ 166.00, 156.54, 149.21, 149.10, 142.56, 137.44, 135.28, 132.04, 131.83, 131.13, 130.13, 128.71, 125.64, 123.72, 120.37, 119.12, 112.73, 112.54, 77.34, 77.02, 76.71, 64.65, 64.37, 61.12, 38.89, 14.70, 14.55, 14.37.

Ethyl3-chloro-5-(5-chloro-3-(N-(4-ethoxy-3-methoxyphenyl)-Nmethylsulfamoyl) thiophene-2-carboxamido)benzoate (**CL3-3**)

This compound was obtained using procedures detailed in our previous publication.[2]

^{1}H NMR (400 MHz, $CDCl_3$) δ 10.00 (s, 1H), 7.72 (p, *J* = 2.0 Hz, 2H), 7.68 (t, *J* = 1.8 Hz, 1H), 7.34 (s, 1H), 6.66 (d, *J* = 2.1 Hz, 1H), 6.56 – 6.53 (m, 2H), 4.38 (q, *J* = 7.1 Hz, 2H), 3.75 (q, *J* = 7.0 Hz, 2H), 3.71 (s, 3H), 1.41 (t, *J* = 7.1 Hz, 3H), 1.31 (t, *J* = 7.0 Hz, 3H). ^{13}C

NMR (101 MHz, $CDCl_3$) δ 164.86, 156.70, 149.58, 148.95, 141.95, 138.42, 135.73, 134.70, 132.33, 132.00, 131.94, 130.24, 125.37, 123.18, 118.67, 118.29, 111.80, 111.14, 77.34, 77.02, 76.70, 64.24, 61.54, 55.91, 38.87, 14.51, 14.31.

Spectra

^{1}H NMR of **MK9**

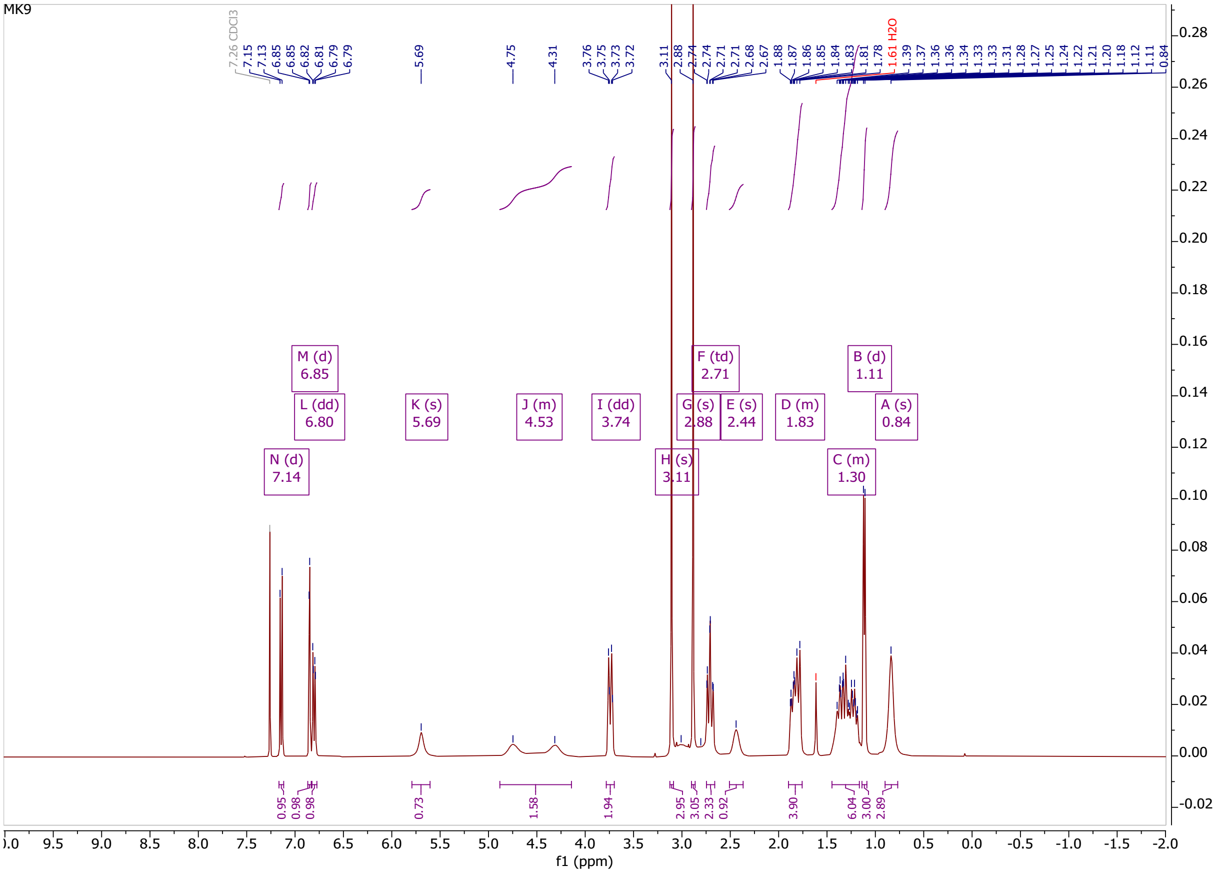

^{13}C NMR of **MK9**

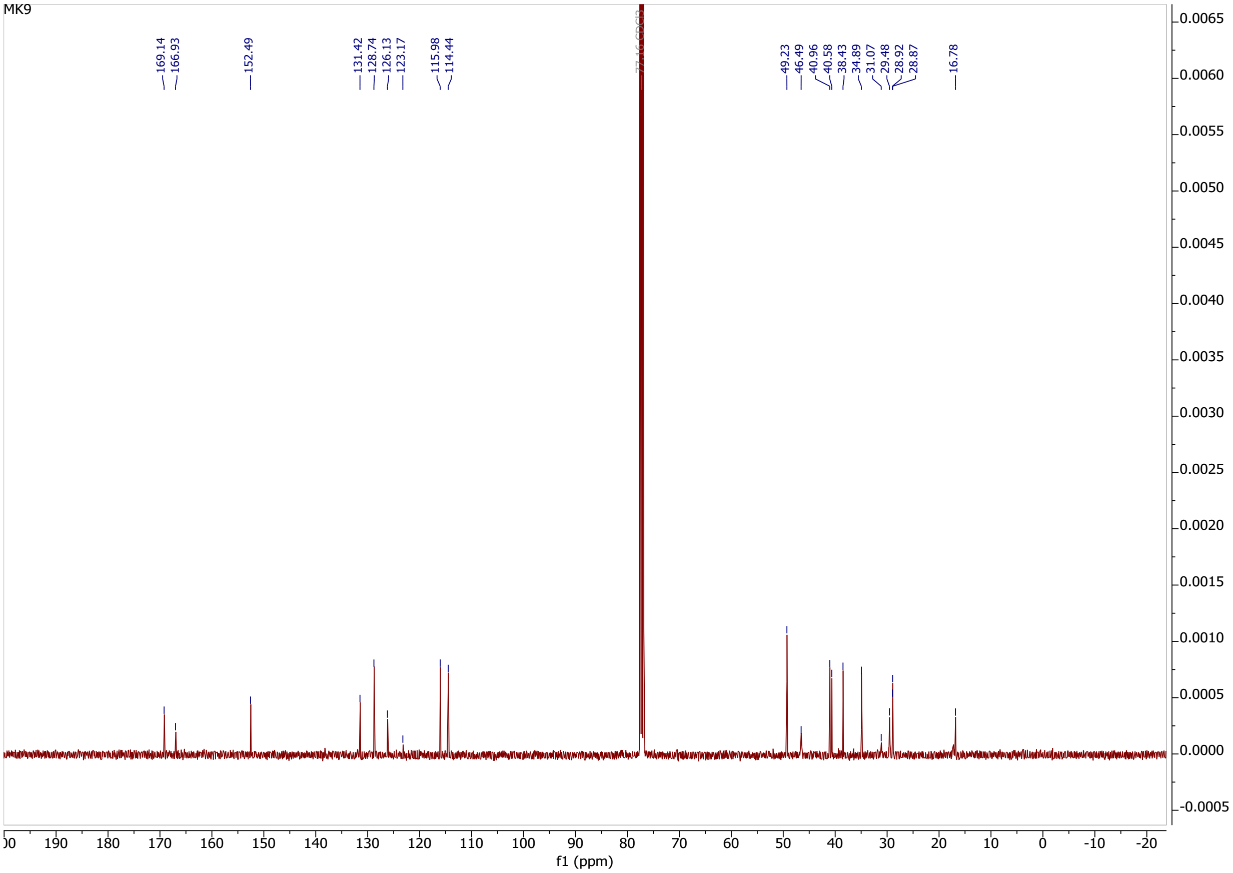

^{1}H NMR of **CL2-57**

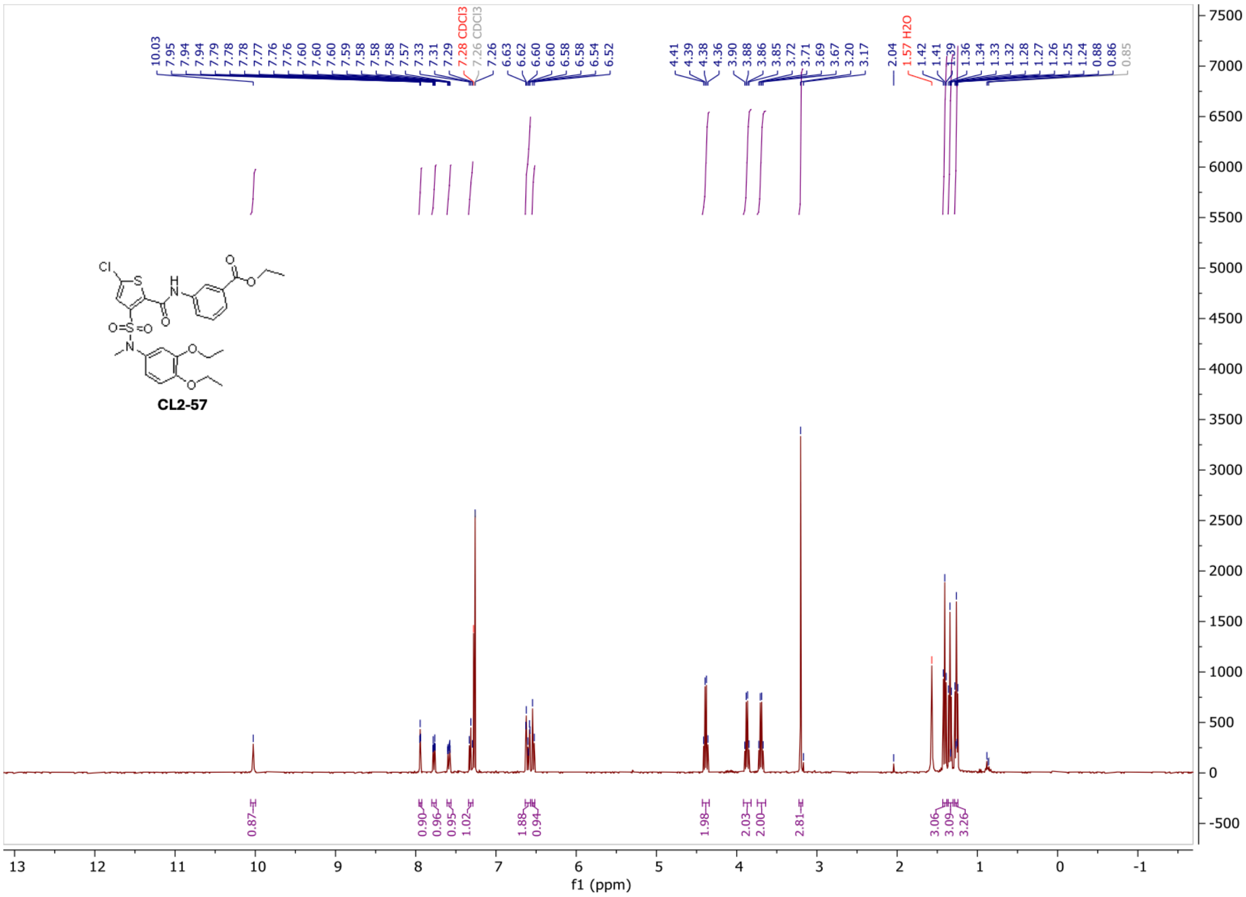

^{13}C NMR of **CL2-57**

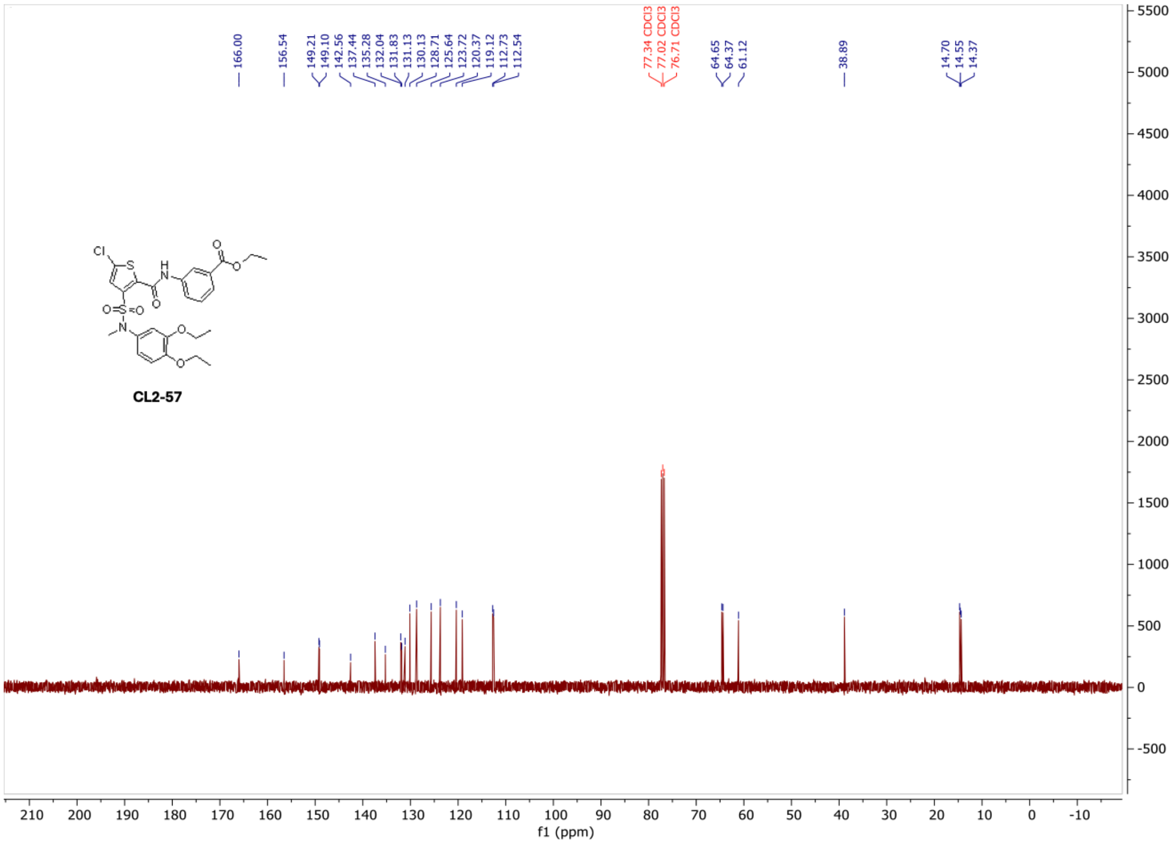

^{1}H NMR of **CL3-3**

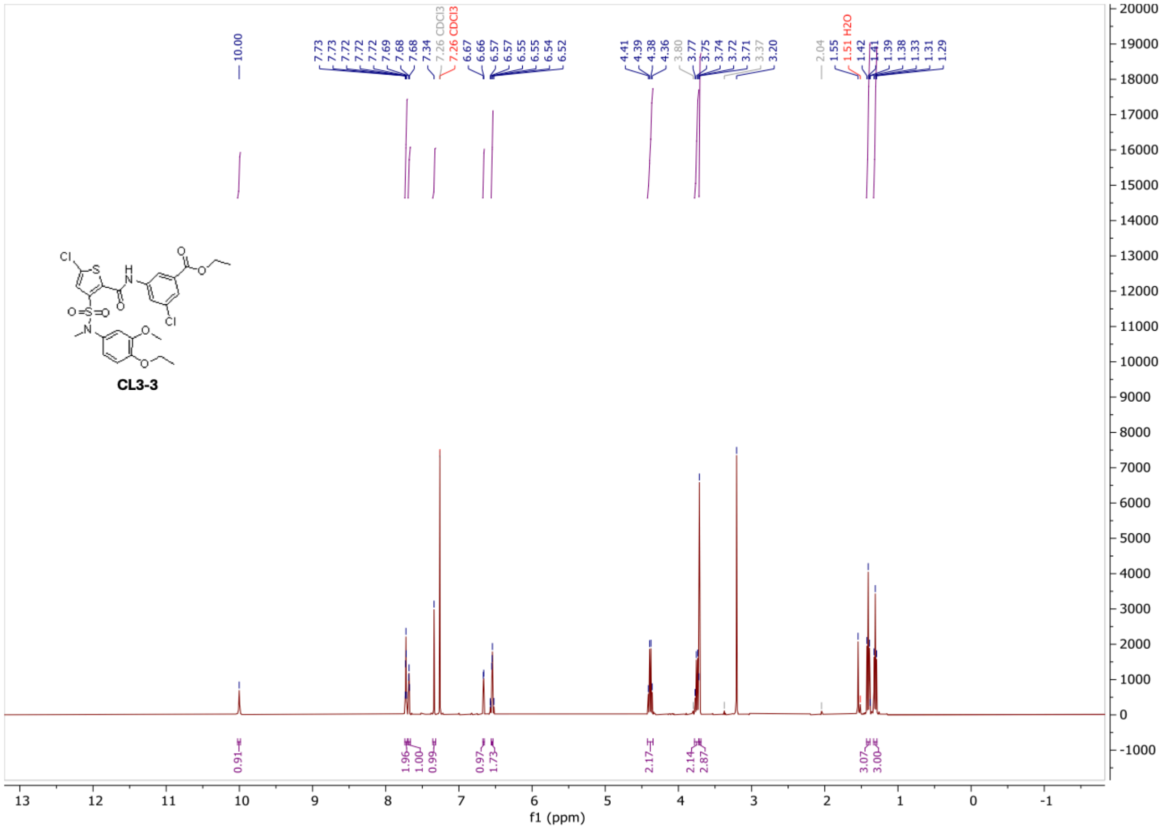

CL3-3

^{13}C NMR of **CL3-3**

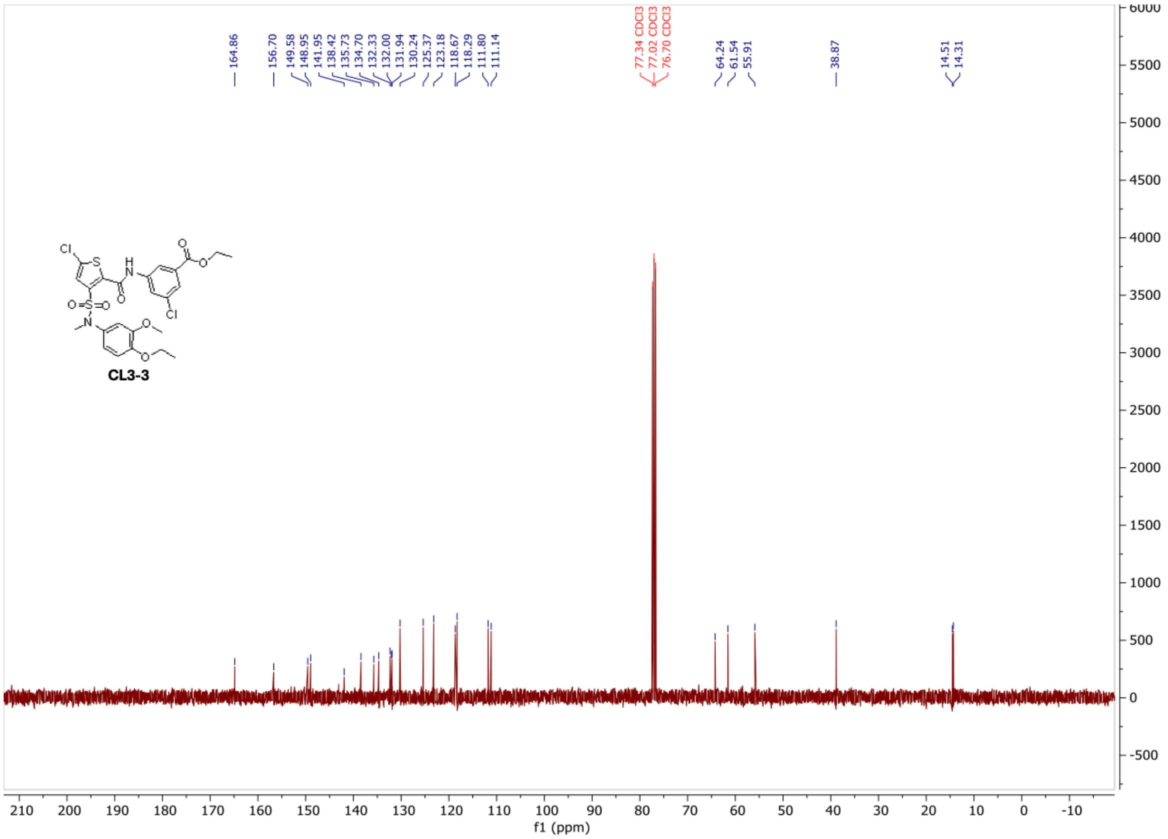

(1) Stachel, S. J.; Zerbinatti, C.; Rudd, M. T.; Cosden, M.; Suon, S.; Nanda, K. K.; Wessner, K.; DiMuzio, J.; Maxwell, J.; Wu, Z.; et al. Identification and in Vivo Evaluation of Liver X Receptor β-Selective Agonists for the Potential Treatment of Alzheimer's Disease. *Journal of Medicinal Chemistry* **2016**, *59* (7), 3489-3498. DOI: 10.1021/acs.jmedchem.6b00176.
(2) Velma, G. R.; Laham, M. S.; Lewandowski, C.; Valencia-Olvera, A. C.; Balu, D.; Moore, A.; Ackerman-Berrier, M.; Rychetsky, P.; Penton, C.; Musku, S. R.; et al. Nonlipogenic ABCA1 Inducers (NLAI) for Alzheimer's Disease Validated in a Mouse Model Expressing Human APOE3/APOE4. *Journal of Medicinal Chemistry* **2024**, *67* (17), 15061-15079. DOI: 10.1021/acs.jmedchem.4c00733.
